# Scale-up of community-based malaria control can be achieved without degrading community health workers' service quality: the Village Malaria Worker project in Cambodia

**DOI:** 10.1186/1475-2875-11-4

**Published:** 2012-01-04

**Authors:** Junko Yasuoka, Krishna C Poudel, Po Ly, Chea Nguon, Duong Socheat, Masamine Jimba

**Affiliations:** 1 Department of Community and Global Health, The University of Tokyo, 7-3-1 Hongo, Bunkyo-ku, Tokyo 113-0033, Japan; 2 National Centre for Parasitology, Entomology and Malaria Control, 372 Monivong Boulevard, Phnom Penh, Cambodia

**Keywords:** Scale-up, Malaria control, Community health workers, Service quality, Cambodia

## Abstract

**Background:**

Malaria control has been scaled up in many developing countries in their efforts to achieve the Millennium Development Goals. Cambodia recently scaled up their Village Malaria Worker (VMW) project by substantially increasing the number of VMWs and expanding the project's health services to include treatment of fever, diarrhoea, and Acute Respiratory Infections (ARI) in children under five. This study examined if the scale-up interfered with VMWs' service quality, actions, and knowledge of malaria control, and analysed VMWs' overall achievements and perceptions of the newly added health services.

**Methods:**

Structured interviews were conducted pre scale-up in February-March 2008 with 251 VMWs and post scale-up in July-August 2010 with 252 VMWs. Comparing the pre and post scale-up survey results (n = 195), changes were examined in terms of VMWs' 1) service quality, 2) malaria prevention and vector control actions, and 3) knowledge of malaria epidemiology and vector ecology. In addition, VMWs' newly added health services were descriptively analysed based on the post scale-up survey (n = 252).

**Results:**

VMWs' service quality and actions significantly improved overall during the scale-up of the VMW project (mean index score: +0.805, *p *< 0.001; +2.923, *p *< 0.001; respectively). Although most of knowledge areas also showed significant improvement (between +0.256 and +0.499, *p *< 0.001), less than half (10.3%-47.7%) of the VMWs correctly answered a set of questions on malaria epidemiology and vector ecology, even in the post scale-up survey. About 70% of the respondents reported that their health services to control malaria remained the same or that they were more active after the scale-up. Two-thirds (66.3%) had become more enthusiastic about serving as a VMW since the scale-up, and all but one respondent reported being willing to continue the new services.

**Conclusions:**

The Cambodian experience clearly demonstrated that a nationwide scale-up of community-based malaria control can be achieved without degrading community health workers' service quality. The government's strategy to expand VMWs' health services, while providing sufficient training to maintain the quality of their original malaria control services, could have contributed to the improvement of VMW's service quality, actions, and knowledge in spite of the rapid scale-up of the project.

## Background

There is a growing consensus that it is critical to scale-up national malaria control programmes in affected countries to meet the Millennium Development Goal (MDG) target for reducing malaria [1]. Since 2005, the concept of Scale-Up for Impact (SUFI) has also been endorsed by the Roll Back Malaria Partnership. It aims at achieving widespread coverage of a set of preventive and treatment interventions that will lead to a dramatic reduction in the global disease burden that malaria poses [2,3]. Rapid and accelerated coverage increase is considered to achieve a substantial burden reduction and added benefit from accelerated scale-up, compared to gradual incremental coverage increase [1].

Malaria control has been scaled up in a number of low-and middle-income countries during the last several years [4-8], and its effectiveness of reducing malaria mortality and morbidity has been demonstrated in several studies [3,5]. In Ethiopia and Rwanda, long-lasting insecticidal nets and artemisinin-based combination therapy that had been distributed nationwide by 2007 reduced in-patient malaria cases and deaths in children under five by > 50% [8]. Zambia has reduced child parasitaemia and severe malaria by > 50% and child mortality by > 30% through scaling up a package of malaria interventions [9]. According to a study that explored the relationship between all-cause child mortality and malaria mortality in sub-Saharan Africa, if malaria control interventions were scaled up to achieve 70% coverage, malaria mortality could be reduced by 50% [2].

Scale-up can only be achieved by substantially increasing the delivery of malaria interventions and requires an adequate number of well-trained human resources [1]. Therefore, community health workers have come to receive much attention in promoting scale-up of disease control and achieving MDGs [10-12]. However, there is a massive global shortage and mal-distribution of health workers [13-15] and hence an urgent need of task shifting by making more efficient use of the existing cadres and to train new cadres to expand the health workforce [11,16,17]. In order to approach the MDGs, sub-Saharan African countries need to triple their health workers by adding one million workers through retention, recruitment, and training [14]. All Southeast Asian countries also struggle with the mal-distribution of health workers, especially in rural areas [15].

To strengthen the national malaria control programme through training more community health workers, Cambodia's National Centre for Parasitology, Entomology and Malaria Control (CNM) recently scaled up their Village Malaria Worker (VMW) project. This project was initiated in a remote province in 2001 and gradually expanded until 2008, identifying malaria-prone villages, where two VMWs were selected through community consensus in each village. Trained VMWs are supposed to perform rapid diagnostic tests (RDTs) on any villager suspected of having malaria, to provide anti-malarials for test-positive cases, according to the national guidelines, and to refer severe cases to hospitals. They are also encouraged to conduct active case detection, record fever cases and positive RDT results, follow up patients, and provide information on malaria preventive measures to their villagers. Their services are directly supervised by the CNM staff in two ways: 1) check VMWs' records and resupply RDT kits and medications at monthly meetings held at health centers in each region, and 2) visit each VMW village twice a year to monitor VMW activities and observe their relationship and communication with villagers [18].

The scale-up took place in 2009 with technical support from the World Health Organization (WHO) and financial support from three international donors. The VMW project was scaled up in three dimensions: the number of VMWs was increased, the number of villages with VMWs was increased, and the range of the project's health services was expanded. Approximately 2,000 new VMWs were trained in malaria prevention, diagnosis, and treatment. At the same time, the number of villages with VMWs more than quadrupled from 315 in seven provinces to 1394 in 17 provinces.

**Figure 1:**
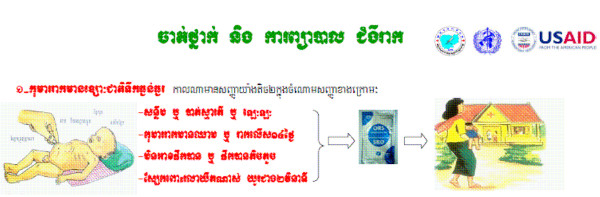
**Sample artwork from VMW training material for diarrhoea treatment of children under five**.

Furthermore, the health services provided by all the VMWs in original 315 villages, who had already been trained and started malaria control services by 2008, were expanded. These VMWs participated in a newly developed three-day training programme, which covered fever, diarrhoea, and ARI case management and prevention for children under five (Figure 1), in addition to a two-day refreshers' training programme on malaria control. Most of the VMWs initiated their new health services within a month or two, utilizing the training material and medicine supplied by the CNM.

Expanding the VMWs' health services was an attempt to merge the VMW scheme with the country's Integrated Management of Childhood Illnesses (IMCI) [19]. Integration of the fever, diarrhea, and ARI interventions became possible in collaboration with the IMCI programme and the National Programme for ARI, Diarrhoea and Cholera under the Ministry of Health (MoH). The necessary medications (paracetamol, oral rehydration salts (ORS), zinc, and cotrimoxazol) were procured and supplied through the MoH, utilizing funding from the WHO. This attempt reflects a recent recommendation made by several researchers to link a vertical approach that promotes disease-specific control programmes with a horizontal approach that focuses on strengthening the overall structure and functions of a health system in order to make a "diagonal approach" that could impact health systems and improve overall health status [20-23].

This study examined whether the scale-up of the VMW project with the additional health services interfered with the VMWs' original services to control malaria. By comparing VMWs' service quality, actions, and knowledge of malaria control before and after the scale-up, changes in their performance were detected, and determinants of these changes were identified. This study also descriptively analysed VMWs' overall achievements, perceptions, and knowledge regarding the additional health services they performed.

## Methods

### Data collection

This study was conducted in 7 remote provinces of Cambodia where 315 VMW villages had been established prior to 2008: Rattanakiri, Kratie, Mondurkiri, Stung Treng, Kampong Thom, Kampot, and Preah Vehear. Pre scale-up survey was conducted from February-March, 2008, as described in a previous paper [18]. Post scale-up data was conducted from July-August, 2010. In both surveys, face-to-face interviews were conducted by trained surveyors, targeting the head VMW who leads the malaria control activities in each of the 315 VMW villages.

In the pre scale-up survey, a structured questionnaire with 46 questions addressed the VMWs' 1) socio-demographic characteristics, 2) service quality, 3) actions to prevent malaria and control vectors, and 4) knowledge of malaria epidemiology and vector ecology [18]. The post scale-up survey questionnaire had 16 additional questions to examine VMWs' new health services to treat fever, diarrhoea, and ARI in children under five in terms of 1) the VMWs' overall achievements and perceptions of the new services, 2) changes in VMWs' workload for malaria control since service expansion, and 3) accuracy of VMWs' knowledge of treatment. Both questionnaires were developed in English, translated into Khmer by local malaria experts, back-translated to English by another expert, and piloted on VMWs in Kampot province.

### Measures: quality, action, and knowledge indices

As described in detail in our previous paper, a "Quality index", an "Action index", and "Knowledge index" were developed, based on respondents' answers to the survey questions [18]. Briefly, the Quality index measured the quality of VMWs' services for malaria control, focusing on five items: active detection, diagnosis and treatment, prescription of anti-malarials, follow-up of patients, and dissemination of preventive measures. The score for each item was calculated as [total points divided by maximum points], so that each item was given a maximum of 1 point, and was added up to create the index. The Action index was developed to quantify the different malaria preventive and vector control measures the VMWs took. Each measure was given either 1 or 2 points, depending on its effectiveness and frequency. The Knowledge index measured quantified VMWs' understanding of malaria epidemiology and vector ecology: malaria symptoms, malaria transmission, vector species, vector active time, vector development time, breeding places, and natural enemies.

### Newly added services for fever, diarrhoea, and ARI treatment

VMWs were asked about how long they had been offering the new services and the number of diarrhoeal and ARI patients they treated per month. Three questions assessed changes in their workload since the service expansion, especially on the perceived change in the activeness for malaria control and in their enthusiasm about serving as a VMW. Two questions addressed their willingness to continue the new services and support needed, and six questions examined whether knowledge of fever, diarrhoea, and ARI treatment was accurate, focusing on the prescription of medicine (correct dosage of paracetamol, ORS, and cotrimoxazole, and how to prescribe them) and the need of referring patients to a hospital, depending on patients' age and severity.

### Data management, statistical analyses, and ethical considerations

Data from the 195 VMWs who participated in both the pre and post scale-up surveys were used to detect any changes in their malaria control services before and after the scale-up of the VMW project. Paired t-tests were conducted for each index to examine the changes in VMWs' service quality, actions, and knowledge. McNemar's test was conducted for each item that created the indices, to test if there were any significant differences between pre and post scale-up survey results. To identify determinants of the changes in VMWs' service quality and actions, multiple linear regression analyses were run with 15 independent variables: eight socio-demographic factors and seven variables regarding the change in each knowledge area described above. Using the post scale-up data from VMWs, descriptive analyses were conducted regarding the new health services. All data analyses were done using STATA/SE version 11.

Informed consent was obtained from all participants for the interviews in both surveys. Participation was voluntary, and confidentiality was secured. The study protocol, consent forms, and survey questionnaires were approved by the Ethical Committee of the University of Tokyo. They were also reviewed by the CNM Institutional Review Board, National Ethics Committee for Health Research, Cambodia, and were exempted from the ethical procedure.

## Results

### Socio-demographic characteristics

Out of 315 VMWs, 195 (62%) took part in both the pre and post scale-up surveys, and 252 (80%, including the 195) participated in the post scale-up survey (Table 1). At pre scale-up, respondents had served as VMWs for about 3 years, and most of them attended the VMW training or refresher training on malaria control between 1 and 1.5 years ago. At post scale-up, respondents had served as VMWs for about 6 years, and all of them had attended trainings for malaria control as well as fever, diarrhoea, and ARI treatment 7 or 8 months ago.

**Table 1:** Selected socio-demographic characteristics of the study population

Characteristics	Pre and post scale-up survey participants (n = 195)				Post scale-up survey participants (n = 252)			
	
	Mean	SD	Number	%Total	Mean	SD	Number	%Total
Age	38.0	12.4			37.2	12.2		
Education (final grade)	3.7	2.7			3.6	2.7		
Gender*								
Male			160	82.1			203	80.6
Female			35	17.9			47	18.7
Occupation								
Farmer			187	95.9			242	96.0
Other			8	4.1			10	4.0
Region								
Mountainous			118	60.5			157	62.3
Other			77	39.5			95	37.7
Ethnicity*								
Khmer			64	32.8			75	29.8
Other			131	67.2			161	63.9
VMW career (months): pre scale-up	40.2	15.2			N/A	N/A		
VMW career (months): post scale-up	71.0	8.3			69.1	12.3		
Reason for becoming VMW								
Recommended by villagers			100	51.3			102	40.5
Interested in malaria treatment/prevention			95	48.7			150	59.5
Most recent VMW training attended (months ago): pre scale-up	15.6	5.0			N/A	N/A		
Most recent VMW training attended (months ago): post scale-up	7.5	0.5			7.5	0.6		
Most recent training for fever, diarrhoea, ARI treatment attended (months ago)	7.5	0.5			7.5	0.6		

*2 missing data on gender and 16 missing data on ethnicity in post scale-up survey

### Changes in quality, action, and knowledge indices

Both VMWs' service quality and actions for malaria prevention and vector control significantly improved during the scale-up of the VMW project (mean index score: +0.805, *p *< 0.001; +2.923, *p *< 0.001; respectively) (Table 2). Most knowledge areas of malaria epidemiology and vector ecology also significantly improved (between +0.256 and +0.499, *p *< 0.001). Knowledge of vector active time did not improve significantly (+0.015, *p *= 0.090), but the mean knowledge score on the topic (0.979 out of 1) was already high in the pre scale-up survey.

**Table 2:** Changes in indices to measure VMWs' service quality, actions, and knowledge (n = 195)

Indices and their items	Number of items in index	Maximum possible score	Reliability (Chronbach's alpha)	Pre scale-up		Post scale-up		Change in mean	Paired t-test *p*-value*
						
				Mean	SD	Mean	SD		
Service quality	5	5	0.822	3.258	0.900	4.063	0.440	0.805	< 0.001
Active detection									
Diagnosis and treatment									
Prescription of anti-malarials									
Follow-up									
Dissemination of preventive measures									

Actions	2	23	0.813	13.159	4.217	16.082	2.140	2.923	< 0.001
Malaria preventive measures									
Vector control measures									

Knowledge									
Malaria symptom	5	1	0.591	0.568	0.223	0.838	0.183	0.270	< 0.001
Malaria transmission	6	1	0.797	0.585	0.317	0.841	0.199	0.256	< 0.001
Vector natural enemies	4	1	0.783	0.114	0.244	0.613	0.344	0.499	< 0.001
Vector breeding places	4	1	0.744	0.347	0.321	0.751	0.281	0.404	< 0.001
Vector development time	1	1	N/A	0.072	0.259	0.354	0.479	0.282	< 0.001
Vector species	6	1	0.856	0.329	0.294	0.604	0.197	0.275	< 0.001
Vector active time	1	1	N/A	0.979	0.142	0.995	0.072	0.015	0.090

*One-tailed t-test *p*-value

### Changes in service quality

Widespread significant improvement was observed in most items related to service quality (Table 3). To diagnose malaria, more measures were taken, in addition to using RDTs, at the time of the post scale-up survey. In the post scale-up survey, all VMWs responded that they always prescribed artesunate and mefloquine (A+M) to treat those with positive RDT results (+2.1%, *p *= 0.046). When prescribing anti-malarials, more VMWs explained about the importance of compliance, in addition to the appropriate dosage after the scale-up (+25.6%, *p *< 0.001). VMWs' understanding of the possibility of compliance failure causing or spreading drug resistance significantly improved (+85.6%, *p *< 0.001), as did their understanding of other issues regarding the prescription of anti-malarials (between +17.9% and +28.2%, *p *< 0.001). The percentage of VMWs who followed up patients to make sure that they recovered from malaria significantly increased (+34.4%, *p *< 0.001). Dissemination of a variety of vector control measures improved, especially about covering water jars/tanks (+39.5%, *p *< 0.001) and filling in water pools (+25.6%, *p *< 0.001). Active detection of malaria patients was the only item that declined (-18.5%, *p *< 0.001).

**Table 3:** Changes in VMWs' service quality, actions, and knowledge (n = 195)

		Pre scale-up		Post scale-up		Absolute change	*p*-value
				
		n	%	n	%	%	
**Service quality**							

Active detection	Visit villagers to find malaria patients (Regularly)	58	29.7	22	11.3	-18.5	<0.001

Diagnosis and treatment	Take body temperature (Always)	53	27.2	141	72.3	45.1	<0.001
	Observe symptoms (Always)	101	51.8	142	72.8	21.0	<0.001
	Ask symptoms from family (Always)	56	28.7	89	45.6	16.9	<0.001
	Prescribe A+M to those who had positive RDT results (Always)	191	97.9	195	100.0	2.1	0.046
	Use RDTs (Always)	194	99.5	194	99.5	0.0	1.000

Prescription of anti-malarials	Explain about the importance of compliance (Always)	116	59.5	166	85.1	25.6	<0.001
	Explain about dosage (Always)	193	99.0	195	100.0	1.0	0.157
	Compliance failure can cause/spread drug resistance	13	6.7	180	92.3	85.6	<0.001
	Inappropriate to save tablets for next infection	137	70.3	192	98.5	28.2	<0.001
	Inappropriate to save tablets to treat other people's malaria	144	73.8	192	98.5	24.6	<0.001
	Compliance failure can result in incomplete treatment	146	74.9	181	92.8	17.9	<0.001

Follow-up	Make home visits or ask patients' family to check if patients recovered (Always)	40	20.5	107	54.9	34.4	<0.001

Dissemination of preventive measures	Cover water jars/tanks	113	57.9	190	97.4	39.5	<0.001
	Fill in water pools	141	72.3	191	97.9	25.6	<0.001
	Spray house	21	10.8	66	33.8	23.1	<0.001
	Wear long-sleeve shirts/pants	154	79.0	194	99.5	20.5	<0.001
	Bring hammock nets to forest	163	83.6	194	99.5	15.9	<0.001
	Use mosquito coils	24	12.3	52	26.7	14.4	<0.001
	Clear bush around house	169	86.7	195	100.0	13.3	<0.001
	Never told not to come close to malaria patients	156	80.0	182	93.3	13.3	<0.001
	Sleep under bednets	183	93.8	195	100.0	6.2	0.001
	Never told not to share utensils with malaria patients	166	85.1	178	91.3	6.2	0.070

**Actions for malaria prevention and vector control**							

Malaria preventive measures	Come back home before dawn	173	88.7	194	99.5	10.8	<0.001
	Bring hammock nets to the forest	170	87.2	190	97.4	10.3	<0.001
	Sleep under bednets at home	187	95.9	195	100.0	4.1	0.005
	Wear long-sleeved shirts/pants	187	95.9	195	100.0	4.1	0.005
	Refrain from going to the forest	169	86.7	72	36.9	-49.7	<0.001

Vector control measures	Seal holes/cracks on walls/ceilings	16	8.2	90	46.2	37.9	<0.001
	Kill mosquitoes by hands	118	60.5	179	91.8	31.3	<0.001
	Cover water jars/tanks	138	70.8	192	98.5	27.7	<0.001
	Spray house	7	3.6	48	24.6	21.0	<0.001
	Use mosquito coils	8	4.1	42	21.5	17.4	<0.001
	Fill in water pools	162	83.1	193	99.0	15.9	<0.001
	Burn trash around house	171	87.7	194	99.5	11.8	<0.001
	Clear bush around house	171	87.7	190	97.4	9.7	<0.001
	Don't plant flowers/grasses around house	101	51.8	95	48.7	-3.1	0.527

**Knowledge of malaria epidemiology and vector ecology **(correct answers to all questions)							

Malaria symptoms		23	11.8	87	44.6	32.8	<0.001
Malaria transmission		40	20.5	93	47.7	27.2	<0.001
Vector breeding places		12	6.2	75	38.5	32.3	<0.001
Vector development time		14	7.2	69	35.4	28.2	<0.001
Vector natural enemies		4	2.1	40	20.5	18.5	<0.001
Vector species		21	10.8	20	10.3	-0.5	0.862
Vector active time		191	97.9	190	97.4	-0.5	0.180

### Changes in actions

VMWs reported taking more actions to prevent malaria and control vectors in the post scale-up survey than in the pre scale-up survey. Percentage of VMWs who took each malaria preventive measure slightly increased (between +4.1 and +10.8%, *p *< = 0.005), except for refraining from going to the forest (-49.7%, *p *< 0.001). VMWs who took vector control measures such as sealing holes/cracks on the walls/ceilings, covering water jars/tanks, and spraying houses improved significantly (between +21.0% and +37.9%, *p *< 0.001), as did other measures such as using mosquito coils, filling in water pools, and burning trash and clearing bush around houses (between +9.7% and +17.4%, *p *< 0.001).

### Changes in knowledge

Percentage of VMWs who gave correct answers to all questions regarding malaria symptoms, transmission route, vector breeding places, development time, and natural enemies significantly increased (between +18.5% and +32.8%, *p *< 0.001). Nevertheless, only less than half (10.3%-47.7%) of the VMWs were able to correctly answer all questions regarding these topics even in the post scale-up survey, with the exception of one on vector active time, which could be known from experience.

### Determinants of the changes in service quality and actions

The important determinants for the improvement in both VMWs' service quality and actions for malaria prevention and vector control were having a personal interest in malaria control as the reason for becoming a VMW (Beta = 0.739 and 2.490, *p *< 0.001, respectively), having improved knowledge about malaria transmission (Beta = 0.620, *p *< 0.007; Beta = 2.439, *p *< 0.002, respectively), and attending the refresher training earlier (Beta = 0.700, *p *< 0.001; Beta = 1.629, *p *= 0.007, respectively) (Table 4).

**Table 4:** Determinants of the change in VMWs' malaria service quality and in VMWs' actions for malaria prevention and vector control

Change in service quality	Beta coefficient	SE	t	*p*-value
Ethnicity	-0.415	0.141	-2.94	0.004
Reason for becoming VMW	0.739	0.109	6.79	< 0.001
The most recent malaria training	0.700	0.150	4.67	< 0.001
Change in knowledge of malaria transmission	0.620	0.157	3.95	< 0.001
Change in knowledge of vector breeding places	0.458	0.138	3.33	0.001

Change in actions				

Occupation	-4.441	1.386	-3.20	0.002
Reason for becoming VMW	2.490	0.553	4.51	< 0.001
The most recent malaria training	1.629	0.602	2.71	0.007
Change in knowledge of malaria transmission	2.439	0.772	3.16	0.002

(Adjusted R^2 ^= 0.404 and 0.278, respectively, for the best model by backward elimination)

Note: Multiple linear regression analyses were run with 15 independent variables: 8 socio-demographic factors (age, education, gender, occupation, ethnicity, length of VMW career, reasons for becoming VMWs, and the most recent VMW training on malaria control attended; "region" was excluded due to its multicollinearity with ethnicity), and 7 variables regarding the change in each knowledge area

### VMWs' newly added health services for fever, diarrhoea, and ARI treatment

The post scale-up survey results revealed VMWs' overall achievements in the newly added health services to treat fever, diarrhoea, and ARI cases of children under five (Table 5). Nearly all of the respondents (96.9%) began providing the new services shortly after they attended the training during the scale-up. About half (54%) of them treated 1-3 patients with diarrhoea per month, and 30.6% treated 4-6 patients. In general, VMWs treated fewer ARI patients than diarrhoeal patients: about 60% treated 1-3 ARI patients per month, and 19.4% treated 4-6 patients.

**Table 5:** Responses from the post scale-up survey participants (n = 252) about the treatment of fever, diarrhoea, and ARI in children under five

		n	%
Have been offering fever, diarrhoea and ARI treatment services (months)	0-3	2	0.8
	4-5	6	5.2
	6-7	237	94.1
	8	7	2.8

Number of diarrhoeal patients treated/month	0	15	6.0
	1-3	136	54.0
	4-6	77	30.6
	6-9	15	6.0
	10 or more	9	3.6

Number of ARI patients treated/month	0	46	18.3
	1-3	151	59.9
	4-6	49	19.4
	6-9	3	1.2
	10 or more	3	1.2

Change in malaria service since service expansion	More active	2	0.8
	Same	173	68.6
	Less active	77	30.6

Enthusiasm about serving as VMW since service expansion	More enthusiastic	167	66.3
	Same	59	23.4
	Less enthusiastic	26	10.3

Willingness to continue fever, diarrhoea, and ARI treatment services	Yes	251	99.6
	Other	1	0.4

Support needed to improve services for malaria, fever, diarrhoea, and ARI treatment services*	Honorarium/salary	251	99.6
	More advice/supervision from health center	243	96.4
	More advice/supervision from CNM	226	89.7
	More VMWs in the village	80	31.8

Knowledge(correct answer)	Treatment for 4-year-olds with 39.3C	53	21.0
	Referral: one-month-olds with 38.2C	244	96.8
	Treatment for 3-year-olds with diarrhoea > 2 weeks	233	92.5
	Referral: 4-year-olds with bloody diarrhoea	239	94.8
	Treatment for 2-year-olds with ARI symptom	162	64.3
	Referral: 3-month-olds with ARI symptom	58	23.0

*multiple choices ok

In terms of changes in their workload for malaria control since the scale-up, about 70% of the respondents reported that their services for malaria control remained the same or became more active. Two-thirds (66.3%) were more enthusiastic about serving as a VMW since the scale-up. All but one respondent (99.6%) showed willingness to continue the new services.

Accuracy in VMWs' knowledge of fever, diarrhoea, and ARI treatment were examined by a set of questions that considered patients' age and the severity of their symptoms. In general, VMWs correctly answered questions regarding extreme cases, such as a one-month old fever case (96.8%) and a child with bloody diarrhoea (94.8%). However, when it came to details about prescriptions, for example the number of paracetamol or cotrimoxazole tablets to prescribe and how to prescribe them, the answers were often inaccurate (21.0% and 64.3%, respectively).

## Discussion

This study found that community-based malaria control can be scaled up without degrading the quality of the health services that community health workers provide. Cambodian VMWs' service quality and actions for malaria prevention and vector control significantly improved during the scale-up of the VMW project. Most areas of knowledge on malaria epidemiology and vector ecology also showed significant improvement in the post scale-up survey.

This substantial improvement observed could be attributed to both VMWs accumulated experiences and gaining more knowledge through every day practices, as well as the training programmes carried out with the scale-up, which not only covered newly added health services, but also reviewed their original health services to control malaria. VMWs had at least 30 more months of experience between the pre and post scale-up surveys. It is clear from monthly reports they submitted to the CNM that they diagnosed and treated malaria cases throughout the year. There is little doubt that their accumulated experiences contributed to the improvement in service quality, actions, and knowledge. This is supported by our previous study, in which a longer VMW career was associated with better service quality [18].

Another factor that could have been effective in improving their original health services is the training programmes organized by the CNM during the scale-up, which covered both new and original health services. Several previous studies have stressed the importance of leadership from national governments and thorough training of community health workers for successful task shifting. To make the task shifting successful in the long run, governments should make serious political and financial commitments to implement the process, ensure adequate resources, and support training activities [17,24]. It is likely that negative unintended consequences of the scale-up have been prevented by the CNM's direct supervision of the VMWs, which has been continued since the beginning of the VMW project [18], as well as their strategy to give VMWs new tasks, while making sure the quality of the VMWs' malaria control services are maintained.

Despite the overall improvement in VMWs' service quality, actions, and knowledge, some items needed additional attention. The most important among these is VMWs' knowledge of malaria epidemiology and vector ecology. Even in the post scale-up survey, less than half of the VMWs correctly answered a set of questions on most knowledge areas. Since the improvement in knowledge was found to be an important determinant for improvements in service quality and actions, it is recommended that more efforts would be made to improve VMWs' knowledge on these areas either in the training programme or through supervision.

Another important finding of this study was that about 70% of VMWs responded they were just as active in controlling malaria, or even more so, after they began providing additional health services for under-five children. Moreover, all except one indicated that the service expansion was worthwhile by reporting their willingness to continue the new health services. As demonstrated by a previous study describing a successful intervention delivered by community health volunteers in Nepal, it may be possible to reduce the burden of diarrhoea and ARI by training and engaging VMWs to implement community-based case management and prevention [25].

However, VMWs' knowledge about fever, diarrhoea, and ARI treatment has plenty of room for improvement, depending on the topic. Although VMWs were able to correctly answer questions regarding extreme cases, their answers for questions about the details of prescriptions were often inaccurate. VMW training programmes could better be organized in this regard, using more simple terms or messages that can be more easily remembered by VMWs who have limited formal education. Some previous studies have demonstrated the effectiveness of using simplified messages delivered directly to community health workers in achieving better health service coverage [24].

One limitation of the study was that only self-reported data were used to evaluate VMWs' service quality, actions, and knowledge. However, these data were double-checked with VMWs' records in their monthly reports, and information about VMWs' service quality from local health centre staff in the region were also obtained. At the same time, the absence of a comparison group prevents us from being able to draw definitive conclusions about the actual impact of the scale-up on VMWs' service quality, actions, and knowledge. It has yet to be demonstrated, based on the pre and post scale-up comparison, that the scale-up was achieved without affecting the VMWs' health services that had been provided by the VMWs.

Despite the reported effectiveness of malaria control scale-up in reducing malaria mortality and morbidity, many countries have been struggling with scale-up barriers [23]. One economic and epidemiological assessment estimated that the cost for comprehensive malaria control to reduce malaria burden in Africa by 75% by 2015 would average up to US$3.0 billion per year [26]. Another study regarding 27 sub-Saharan African countries demonstrated that recent scale-up of malaria intervention coverage has achieved equal access to health services in some countries (especially with ITNs), but delivery systems in other countries have not reached the most-at-risk poor and rural populations [27]. In order to make sure that malaria control scale-up will reach at-risk populations with limited access to quality health care, countries need strong leadership and governance, timely access to resources, and coordinated efforts from well-trained health workforce who work at the national, district and local levels [1]. Maintaining and improving the quality of health services provided by community health workers could be key to successfully scale-up national malaria control interventions to achieve MDGs.

## Conclusions

The experience in Cambodia has demonstrated that a nationwide scale-up of community-based malaria control can be achieved without degrading the quality of the health services originally provided by community health workers. The CNM's strategy to expand VMWs' health services, while providing sufficient training to maintain the quality of their original malaria control services, could have prevented possible negative impacts of the scale-up on the quality of VMWs' original services. The lessons learnt from this study can be useful for other countries to successfully scale-up their community-based malaria control interventions in their efforts to achieve MDGs.

## Competing interests

The authors declare that they have no competing interests.

## Authors' contributions

JY conceived the study, developed questionnaires, conducted fieldwork, analysed data, and wrote the manuscript. KCP contributed to the study design, conducted fieldwork, and improved the manuscript. PL, CN, and DS supervised fieldwork. MJ monitored the study progress and provided guidance to improve the manuscript. All authors read and approved the final draft.
